# EHEALTH ON FRAILTY: FRAILSURVEY, A RELIABLE SMARTPHONE APPLICATION FOR SELF-ASSESSMENT OF FRAILTY

**DOI:** 10.1093/geroni/igz038.1221

**Published:** 2019-11-08

**Authors:** Luís Midão, Carla Sá, Elisa Marques, Mafalda Duarte, Constança Paúl, João Viana, Elísio Costa

**Affiliations:** 1 ICBAS - Abel Salazar Institute of Biomedical Sciences, University of Porto, Porto, Portugal, Porto, Portugal; 2 CIDESD - Research Center in Sports Sciences, Health Sciences and Human Development, University Institute of Maia, Porto, Portugal, Porto, Portugal; 3 ISAVE – Superior Institute of Health, Amares, Braga, Portugal, ISAVE – Superior Institute of Health, Braga, Portugal; 4 Instituto de Ciências Biomédicas Abel Salazar, Universidade do Porto, Porto, Portugal; 5 UCIBIO/REQUIMTE, PORTO4AGEING - Competences Centre on Active and Healthy Ageing of the University of Porto, Faculty of Pharmacy of the University of Porto, Porto, Portugal, UCIBIO/REQUIMTE, Portugal

## Abstract

Frailty is a clinical syndrome whose signs and symptoms are predictors of health complications, making this a major public health problem. FRAILSURVEY, which was considered a good practice by several entities, is a smartphone application that allows an easy assessment of frailty, available in Portuguese, English and Italian, on both iOS and Android stores. Smartphone-based assessment has been proven to be a useful diagnostic tool for patients, although few applications have demonstrated reliability. With this work we aimed to test the reliability of FRAILSURVEY as a tool to assess frailty, and to study people’s preference on which way to assess frailty. FRAILSURVEY is a questionnaire that comprises two sets of questions: 19 about sociodemographic data, social resources, self-perception of health, nutrition, medication, psycho-social and cognitive status, and time occupation, plus a set of 15 used to assess frailty status, the Groningen Frailty Indicator (GFI). Including 427 older adults in this study, a randomized repeated measures crossover design was employed using the FRAILSURVEY questionnaire both in paper and in application, with a week of interval to reduce carryover effects. Reliability was assessed through the GFI scores obtained by the same person between the paper and application. There were no significant differences between the results on frailty assessment using the questionnaire on both paper and application. A significant correlation was noted in the total group (ICC=.794, P<0.01). Our work shows that FRAILSURVEY is a reliable mobile application for frailty assessment, that can be used by older adults, caregivers, and health professionals.

